# Macroporous Mannitol Granules Produced by Spray Drying and Sacrificial Templating

**DOI:** 10.3390/ma16010025

**Published:** 2022-12-21

**Authors:** Morgane Valentin, Damien Coibion, Bénédicte Vertruyen, Cédric Malherbe, Rudi Cloots, Frédéric Boschini

**Affiliations:** 1 GREEnMat, CESAM Research Unit, University of Liège, 4000 Liège, Belgium; 2 Mass Spectrometry Laboratory, MolSys Research Unit, University of Liège, 4000 Liège, Belgium

**Keywords:** mannitol, spray drying, polystyrene, porosity, templating

## Abstract

In pharmaceutical applications, the porous particles of organic compounds can improve the efficiency of drug delivery, for example into the pulmonary system. We report on the successful preparation of macroporous spherical granules of mannitol using a spray-drying process using polystyrene (PS) beads of ~340 nm diameter as a sacrificial templating agent. An FDA-approved solvent (ethyl acetate) was used to dissolve the PS beads. A combination of infrared spectroscopy and thermogravimetry analysis proved the efficiency of the etching process, provided that enough PS beads were exposed at the granule surface and formed an interconnected network. Using a lab-scale spray dryer and a constant concentration of PS beads, we observed similar granule sizes (~1–3 microns) and different porosity distributions for the mannitol/PS mass ratio ranging from 10:1 to 1:2. When transferred to a pilot-scale spray dryer, the 1:1 mannitol/PS composition resulted in different distributions of granule size and porosity depending on the atomization configuration (two-fluid or rotary nozzle). In all cases, the presence of PS beads in the spray-drying feedstock was found to favor the formation of the α mannitol polymorph and to lead to a small decrease in the mannitol decomposition temperature when heating in an inert atmosphere.

## 1. Introduction

Nowadays, powder engineering is increasingly used in the pharmaceutical field to improve the efficiency of drugs by enhancing their physicochemical, micromeritic and/or pharmacokinetic properties. In this context, porous particles are studied because their low density and/or their high specific surface area can help to control drug delivery [1,2,3] and improve bioavailability [4]. Some porous particles also display improved compaction ability during tablet manufacturing [4]. Porous particles have been reported in the literature for both active pharmaceutical ingredients (API) [5,6,7] and excipients [2,3,4], in addition to many other fields in materials science, such as electrocatalysis [8,9,10,11], gas sensing [12] and other applications [9,13,14,15].

Several of the methods used to prepare low-density porous excipient and/or APIs particles are based on spray drying, a technique where droplets of solution/suspension are converted into a dry powder by the evaporation of the solvent/liquid [16,17,18,19].

The different spray-drying strategies to create porous dried particles differ by the nature of the precursors that will be transformed into pores. In the PulmoSpheres^TM^ technology [5], small emulsion droplets of a less volatile liquid are dispersed in the liquid containing the pharmaceutical substance; the evaporation of these emulsion sub-droplets during the last stage of the spray-drying process creates pores [6,7,17]. Another approach relies on ammonium bicarbonate as a pore-forming agent whose decomposition takes place after the vaporization of the solvent [17,20,21]. The strategy used in the present work involves spherical beads as a sacrificial template that is removed after the spray-drying step. This sacrificial templating approach has been much studied for the preparation of metal oxides or silica with controlled porosity using a polymer template removed by heat treatment [22,23,24,25,26,27,28]. Polystyrene beads are one of the most common sacrificial templates because their diameter can be controlled during the synthesis allowing a broad range of pore sizes [13,14,15,27,28,29].

However, the sacrificial template strategy has been much less studied in the case of porous organic particles [29,30], where the low thermal stability of the host requires that the template be removed by dissolution/etching instead of a heat treatment. In this context, the best-documented system is that of hyaluronic acid combined with polystyrene beads, later dissolved in toluene [29,30].

Here, we focus on mannitol, an excipient that is one of the most popular alternatives to lactose because it offers improved chemical stability and lower intolerance/allergy issues [31,32]. In the context of the Dry Powder Inhaler (DPI) technology, mannitol can act as an API to improve mucus clearance in people living with cystic fibrosis [33], but is more commonly used as a carrier of the API, for example in the treatment of asthma or chronical obstructive pulmonary disease (COPD) [34,35]. The most common DPI delivery strategy relies on fine (<10 µm) and/or coarse (20–100 µm) mannitol particles mixed together and then mixed with the API [36]. As a result, the API is located at the surface of the excipient particles. Co-spraying the API with the excipient would allow for process intensification but corresponds to a different drug delivery profile [16,37,38,39]. Using polystyrene beads as the sacrificial template, a 1:1 mannitol/polystyrene mass ratio would correspond to a maximum apparent density of 0.62 g/cm^3^ for the porous mannitol particles, compared to 1.514 g/cm^3^ for dense mannitol [40]. Porous mannitol granules are therefore expected to travel more efficiently into the pulmonary system.

Because toluene [29,30] is a Class 2 organic solvent suspected of causing irreversible and reversible toxicity [41], we selected ethyl acetate as the solvent to dissolve the polystyrene beads. Indeed, the Hildebrand solubility parameters of ethyl acetate and polystyrene are similar (18.2 and 18.3 MPa^½^, respectively) [42], and ethyl acetate is approved by the Food and Drug Administration (FDA) as a Class 3 solvent [43,44], and by the European regulatory committee for pharmaceutical applications [40,45].

In the present work, our main objective was to investigate the influence of the mass ratio between mannitol and polystyrene beads on the porosity observed after etching in ethyl acetate. The efficiency of the etching was examined through infrared spectroscopy and thermogravimetric analyses. We also report the influence of the mass ratio between mannitol and polystyrene beads on the polymorphism of mannitol. Then, we transferred the synthesis from lab-scale to pilot-scale conditions and considered how this affects the homogeneity of the porosity distribution. These final sections also include preliminary tests on the possibility of preparing formulations of the spray-dried porous mannitol with an API (budesonide) for inhalation therapy.

## 2. Materials and Methods

### 2.1. Materials

Mannitol Pearlitol^®^ was obtained from Rocquette Frères (Lestrem, France). Potassium persulfate and styrene (≥99% purity) were purchased from Sigma-Aldrich. Ethyl acetate (≥99.8% purity) was purchased from VWR Chemicals. MilliQ (Millipore Milli-Q Plus) water was used for all syntheses. Budesonide was supplied by Sicor Societa Italiana Corticosteroidi Srl (Milano, Italy).

### 2.2. Aqueous Suspension of PS Beads

**Figure 1:**
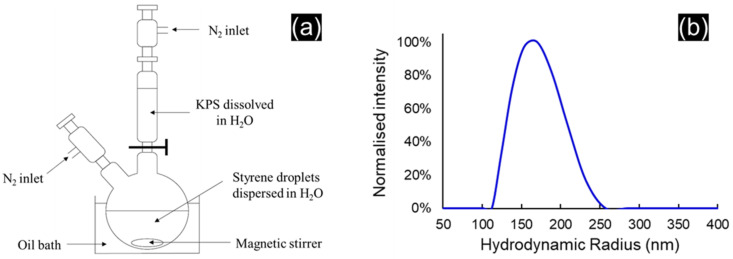
(**a**) Schematic illustration of the apparatus used to produce the PS beads by surfactant-free emulsion polymerization and (**b**) distribution of hydrodynamic radius obtained with dynamic light scattering.

Aqueous suspensions of polystyrene beads were freshly prepared before each series of experiments by surfactant-free emulsion polymerization as described in the literature [46,47]. In a typical synthesis (Figure 1a), 2.72 g of styrene were added to 100 g of milliQ water in a round-bottom flask with a magnetic stirrer rotating at 450 rpm. After N_2_ flushing and heating at 70 °C in an oil bath, 175 mg of potassium persulfate (KPS) initiator dissolved in 25 g of milliQ water was introduced via a dropping funnel. The reaction mixture was maintained under stirring at 450 rpm for 24 h. The as-obtained PS latex with a concentration of 20.18 g/L was used without dilution for the syntheses in the pilot-scale GEA Niro Mobile Minor spray dryer. The latex was diluted by a factor of 6 for the syntheses in the lab-scale Büchi Mini B-191 spray dryer.

### 2.3. Spray Drying of Mannitol/PS Granules

Mannitol/PS granules were obtained by dissolving mannitol Pearlitol^®^ powder in the water-based PS latex and spray drying the suspension. Table 1 provides an overview of the samples: mannitol/PS granules with different mannitol:PS ratios were prepared in the Büchi lab-scale spray dryer while upscaling experiments for the 1:1 ratio were carried out in the pilot-scale Niro spray dryer. The spray-drying parameters are listed in Table 2.

### 2.4. Etching of the PS Beads to Form Porous Mannitol Granules

Etching of the PS beads to produce porous mannitol granules was performed by dispersing 200 mg of mannitol/PS granules in 20 mL of ethyl acetate and stirring for 60 min. The etched granules were then recovered by filtration on a 0.45 µm membrane nylon filter, washing with small volumes of ethyl acetate and drying in an oven at 50 °C for 24 h.

### 2.5. Mannitol-Budesonide Formulations

Formulations containing 0.8% *w*/*w* of API were prepared in a Turbula^®^ mixer (45 rpm for 30 min, Eskens Benelux, Mechelen, Belgium) by mixing about 1 g of porous mannitol granules (excipient) with micronized budesonide (active pharmaceutical ingredient). Cylindrical aluminum containers (3 cm diameter, 3 cm height) were used in order to minimize electrostatic charging.

### 2.6. Characterization Techniques

The hydrodynamic radius distribution of the PS beads was measured using dynamic light scattering (DLS-Viscotek model 802, DLS Viscotek Europe, London, UK) after 100× dilution of the latex. The stability of the latex over 24 h was studied using a Turbiscan^®^. The zeta potential was measured under constant stirring with a DT-1200 zetameter.

The shape and the surface of the granules were examined with a scanning electron microscope (FEI XL30 ESEM-FEG, FEI Eindhoven The Netherlands) operating at an acceleration voltage of 4 keV for mannitol-containing samples and at 15 keV for PS beads. Prior to imaging, a few milligrams of samples were dispersed onto carbon sticky tabs and coated with a gold layer of approximately 20 nm.

Thermal analysis of the granules before and after etching was performed with a TGA-DTA-DSC analyzer (Setaram labsys Evo instrument), previously calibrated using indium, tin, lead, zinc and aluminium fusion points. A total of 5 mg of each sample was heated at 5 °C/min in alumina crucibles under helium atmosphere. The temperatures for the fusion and the decomposition of mannitol reported later in the text were taken as the extremum in the DSC peak (fusion) and the middle of the mass loss in the TG curve (decomposition).

X-ray diffractograms were collected in Bragg–Brentano geometry using a Bruker D8 Twin-Twin diffractometer with a Lynxeye-XET 1D detector, Bruker, Karlsruhe, Germany (Cu-Kα radiation, 2θ = 5–60°, 0.02° step size and 0.1 s/channel step time). The percentages of the α and β mannitol phases were estimated by Rietveld refinement with the TOPAS software, Version 4.2 using the fundamental parameters approach to model the instrumental contribution [48]. The structural models were taken from the PDF4+ database (PDF 00-022-1793 for the α polymorph and PDF 00-022-1797 for the β polymorph).

Attenuated total reflectance infrared (ATR-IR) spectra were recorded on a few milligrams of powders using a Nicolet IS5 with an ID7 ATR accessory (Thermo Scientific) equipped with a diamond crystal.

The homogeneity of the budesonide–mannitol mixing was determined on two aliquots of 150 mg of the mixed powder dissolved in a 40/60 (*v*/*v*) MeOH/water solvent mixture (100 mL). The dosage of budesonide was carried out by an ultra-high performance liquid chromatography UHPLC (Agilent Technologies 1290 infinity) using a reversed-phase column (Acquity CSH C18 1.7 µm, Waters, Milford, USA) coupled to a UV detector for the detection and the quantification of budesonide. The amount of budesonide in the aliquots was determined using a calibration curve with a reference standard.

## 3. Results and Discussion

### 3.1. Preparation of the Aqueous Suspension of PS Beads

**Figure 2:**
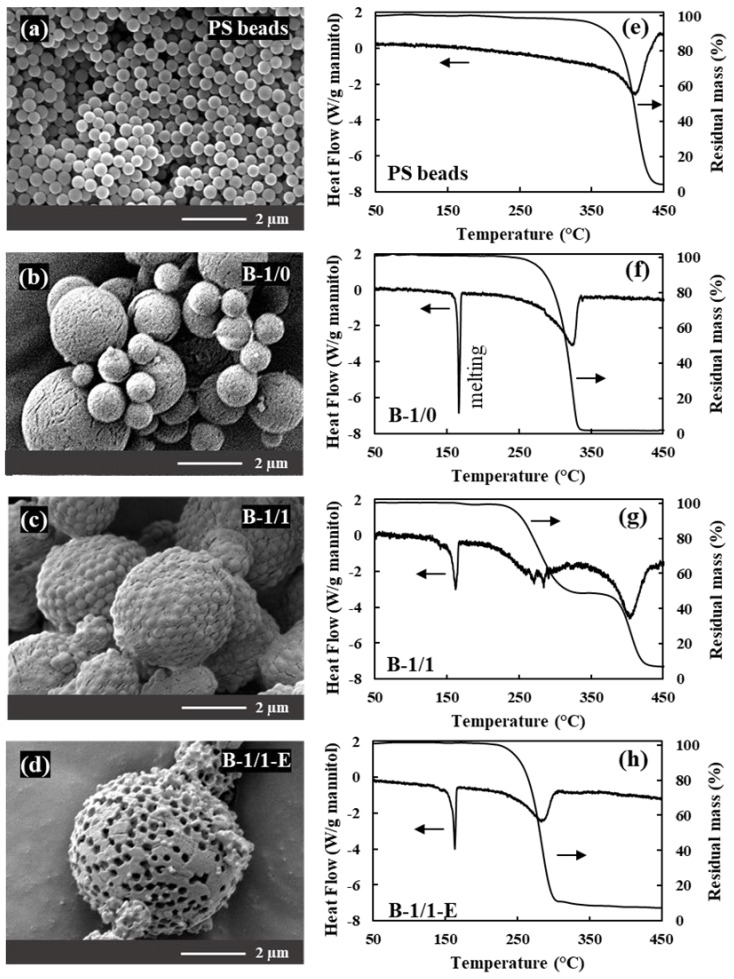
**The** SEM micrographs, TG and DSC curves (5 K/min in helium) for (**a**,**e**) PS beads, (**b**,**f**) spray-dried mannitol (B-1/0), (**c**,**g**) spray-dried granules with a mass ratio of mannitol to PS beads of 1:1 (B-1/1) and (**d**,**h**) the same granules after etching in ethyl acetate (B-1/1-E). The spray-dried samples shown in this figure were obtained with the lab-scale Büchi spray dryer.

Figure 1b shows the radius size distribution of the PS beads measured by dynamic light scattering, revealing a narrow distribution with a diameter of 335 ± 40 µm (mean PS bead diameter ± 3 standard deviations). The SEM micrograph in Figure 2a shows the smooth spherical shape of the PS beads. The zeta potential of −96 mV ensures good dispersion and avoids the aggregation of the beads in the suspension. The migration rate inside the suspension was 0.2 mm/h, confirming the good stability of the suspension of the PS beads.

### 3.2. Preparation of Porous Mannitol Granules by Spray Drying and PS Etching

Figure 2 shows SEM micrographs and thermal analysis curves for (a, e) the PS beads, (b, f) a mannitol-only spray-dried sample and (c, d, g, h) 1:1 mannitol/PS spray-dried granules before and after etching in ethyl acetate. As can be seen in Figure 2c, the 1:1 mannitol/PS granules retain the spherical microstructure observed in the mannitol-only sample (Figure 2b). The possibility of dissolving the polystyrene beads in ethyl acetate without destroying the mannitol network is evidenced by the porosity clearly visible in the etched granules shown in Figure 2d. The dissolution of the PS beads is further confirmed by the absence of any mass loss in the temperature range of the thermal decomposition of the PS (~380 °C–420 °C) in the thermogravimetric curve of the etched granules (Figure 2h, to be compared to Figure 2e,g for the PS beads and the as-sprayed granules, respectively). The thermal events observed at lower temperatures correspond to the fusion of mannitol (160–165 °C, endothermic peak, no mass loss) and the decomposition of mannitol (endothermic peak + mass loss between 250 °C and 330 °C).

### 3.3. Influence of the Mannitol/PS Ratio on the Etching Efficiency and on the Mannitol Polymorphism

**Figure 3:**
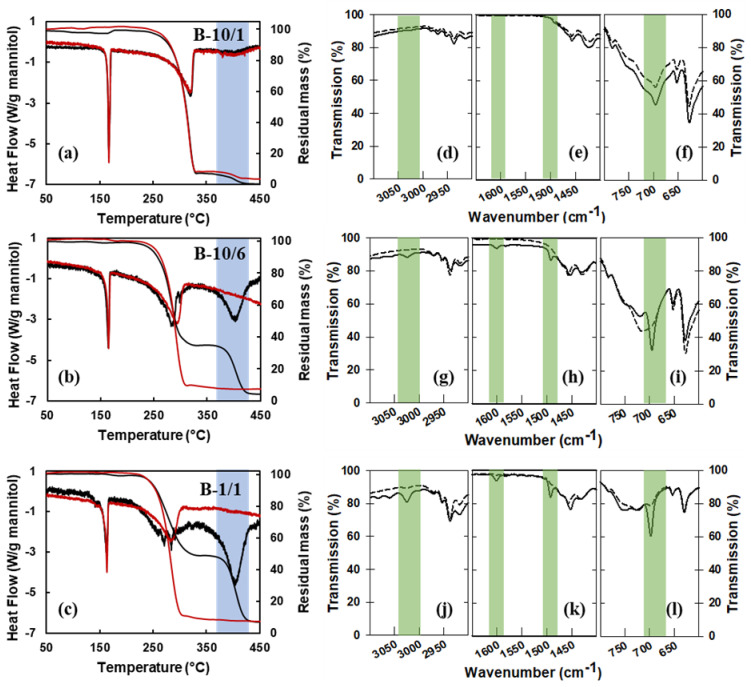
Influence of the M/PS mass ratio on the etching efficiency: (**a**–**c**) TG–DSC curves (5 K/min in helium) of the spray dried granules before etching (black curves) and after etching (red curves); (**d**–**l**) zooms in three regions of the IR spectra of the spray-dried granules before etching (plain curves) and after etching (dashed curves), with the green-shaded areas highlighting characteristic PS vibrations: aromatic C–H stretching at 3025 cm^−1^, aromatic C=C stretching at 1500 cm^−1^ and 1600 cm^−1^ and aromatic C–H bending at 700 cm^−1^ [49]. The spray-dried samples in this figure were obtained with the lab-scale Büchi spray dryer.

**Figure 4:**
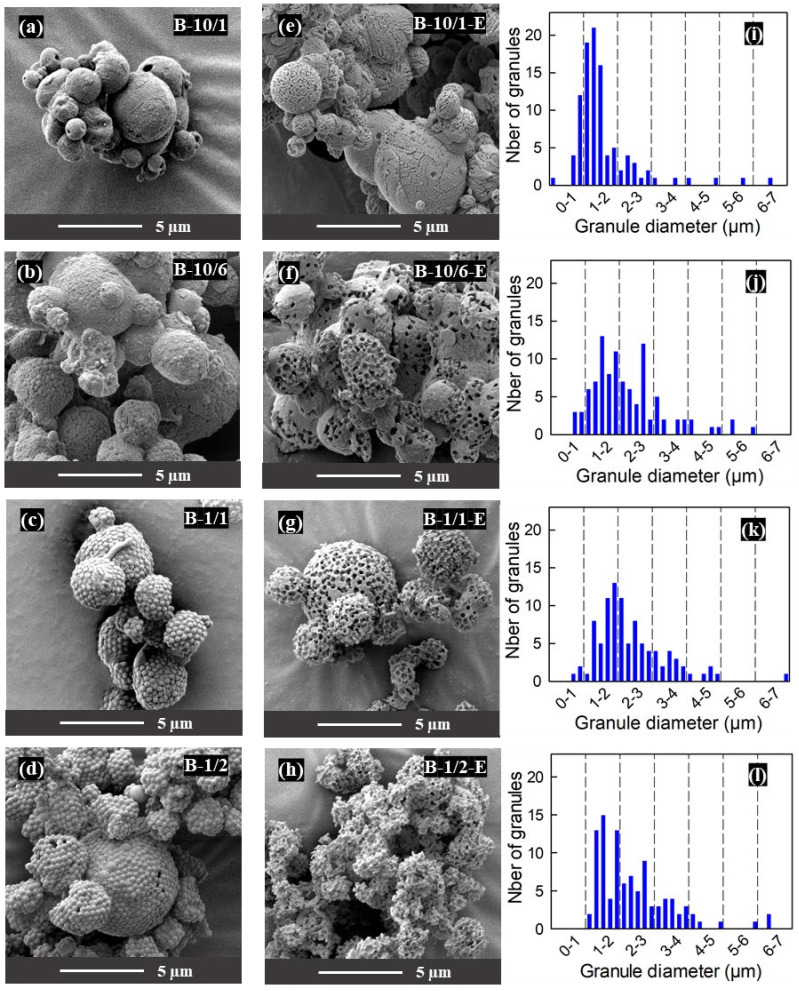
Influence of the M/PS mass ratio on the granule microstructures: (**a**–**d**) SEM micrographs of spray-dried granules before etching; (**e**–**h**) SEM micrographs of spray dried granules after etching; (**i**–**l**) histograms of granule diameters before etching. The spray-dried samples shown in this figure were obtained with the lab-scale Büchi spray dryer.

The left panel of Figure 3 compares the thermal analysis curves of the granules obtained for different mannitol/PS ratios. The data for the 10:6 mannitol/PS ratio (Figure 3b) are similar to those already described in the case of the 1:1 mannitol/PS ratio (shown again in Figure 3c). On the contrary, in the case of the lowest PS content (10:1 mannitol/PS ratio-Figure 3a), the mass loss curves before and after etching reveal that a significant fraction of the PS beads was not dissolved during the etching procedure. This can be explained by the SEM micrographs of sample B-10/1 in Figure 4a,e, where only a few PS beads can be observed at the surface of the as-sprayed granules, suggesting that most of the PS beads are trapped inside the mannitol matrix and cannot be reached by the ethyl acetate solvent.

As a complement to thermal analysis, the same set of samples were characterized by IR spectroscopy, following the approach used by Iskandar et al. (2009) [30] and Nandiyanto and Okuyama (2017) [29] for the hyaluronic acid/PS system. Selected wavenumber ranges of the attenuated total reflectance infrared (ATR-IR) spectra before and after etching are shown in the right-hand part of Figure 3 (see Figure S1 for typical complete spectra). In the ATR configuration, the evanescent IR beam probes only the top ~2 µm of the powder in contact with the diamond crystal and the PS content in the sample with 10:1 mannitol/PS ratio turned out to be too small to be detected, both in the as-sprayed state and after etching. This indicates that the absence of the characteristic PS peaks in the spectra of the etched 10:6 and 1:1 samples should not be considered sufficient proof of a successful etching and needs to be confirmed, as was done here and in Nandiyanto and Okuyama (2017) [29], by thermal analysis results.

**Figure 5:**
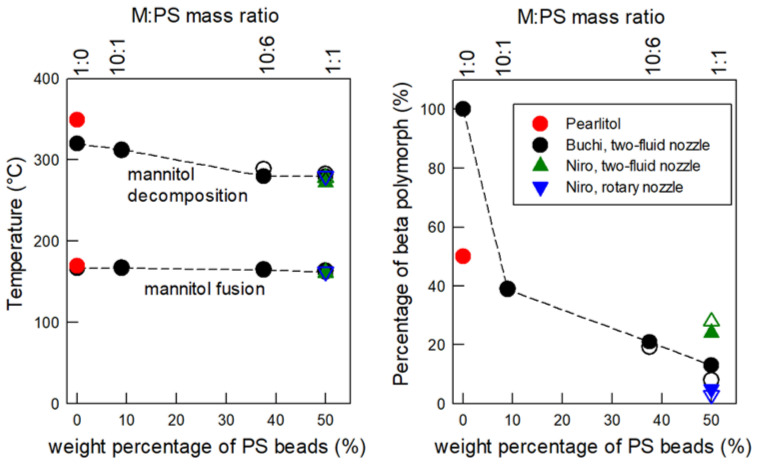
Influence of the M/PS ratio on (**left**) the temperatures of fusion and decomposition of mannitol determined from TGA/DSC curves and (**right**) the percentage of the β polymorph, determined from XRD patterns (see Figure 6) for samples prepared with the lab-scale (Büchi) or the pilot-scale (Niro) spray-driers. The data for the commercial mannitol Pearlitol^®^ is shown for comparison. Empty symbols correspond to the etched samples. The dashed lines are guides for the eye.

The thermal analysis curves in Figure 2 and Figure 3 also provide information about the mannitol component of the granules: Figure 5 (left) plots the temperatures of the fusion and the decomposition of mannitol in the samples prepared with different mannitol/PS ratios. While the fusion temperature is hardly affected, the decomposition temperature decreases by about 40 °C when going from the mannitol-only spray-dried sample to the one prepared with a 1:1 mannitol/PS ratio. The heating rate and the sample masses used for the thermal analysis experiments were the same; therefore, the difference in decomposition temperature can be attributed to actual differences between the samples. The polymorphism was characterized by X-ray diffraction (Figure 5 DRX) and only α and β polymorphs were observed. The percentage of the thermodynamically favored β polymorph is plotted in Figure 5 (right): starting from 100% β polymorph in the mannitol-only spray-dried sample, the β content drops sharply as soon as PS beads are present in the spray-drying feedstock, suggesting that the surface of the PS beads could act as heterogeneous nucleation sites for the kinetically favored α polymorph. Comparing the evolution of the decomposition temperature in Figure 5 (left) and the percentage of β polymorph in Figure 5 (right) shows that polymorphism could be a factor contributing to the evolution of the decomposition temperature but that other effects must play a role. This is confirmed by the data for the commercial Pearlitol mannitol sample, which do not follow the trend. Finally, Figure 5 also shows that neither the decomposition temperature of mannitol nor its polymorphism are much affected by the etching procedure in ethyl acetate.

**Figure 6:**
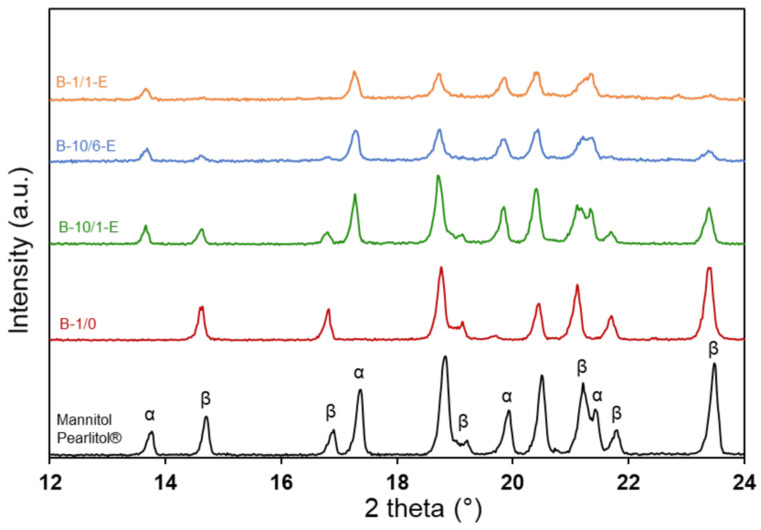
Influence of the M/PS ratio on the polymorphism of mannitol: XRD patterns of the spray-dried granules after etching (lab-scale Büchi spray dryer samples).

### 3.4. Influence of the Mannitol/PS Ratio on the Granule Microstructure

The SEM micrographs in Figure 4 show the evolution of the microstructure as a function of the mannitol/PS ratio. Micrographs of a sample with 1:2 mannitol/PS ratio prepared during preliminary tests are included here although this composition was not further investigated due to the irregular and fragile granule surface structure obtained after etching (Figure 4h). All the other samples are made up from partly agglomerated granules of roughly spherical shape. Table 1 lists the TGA-measured PS contents of these samples and finds them only slightly lower than the nominal ones, indicating that only a minor fraction of the PS beads are lost by attrition in the spray dryer (ending up in the back filter).

The granule size distributions, plotted as histograms of granule diameters measured on SEM micrographs (Figure 4i–l), are similar, with a modest broadening of the distribution towards larger sizes when the PS content increases. This suggests that the granule size is mostly controlled by the constant atomization parameters and the constant concentration of PS beads in this series of experiments (see Table 1). An explanation can be proposed by considering the transport phenomena taking place in the droplets during spray drying [16,50]: the granule size is established when the slowly moving PS beads come into contact with each other because the diffusion of liquid from the center to the surface of the shrinking droplets can no longer compensate for the solvent evaporation from the surface.

While the granule size seems to be dictated by the PS beads, the appearance of the granule surfaces in the SEM micrographs can be related to the mannitol concentration in the droplets, which decreases when the mannitol/PS ratio changes from 10:1 to 1:2 (see Table 1). For a mannitol/PS ratio of 10:1, the surface of the granules is mostly constituted of mannitol, with only a few visible PS beads (Figure 4a). This suggests that the precipitation of mannitol took place when the surface of the granules was still strongly impregnated by liquid. For the other samples, the lower mannitol concentration means that mannitol saturation (and hence precipitation) took place at a later stage of the droplet drying, so that the granules display exposed PS beads on their surfaces. The granules of the sample with an intermediate mannitol/PS ratio of 10:6 display surfaces where both behaviors co-exist (Figure 4b); the homogeneity of the PS bead distribution is much better for the mannitol/PS ratio of 1:1 (Figure 4c), while the highest mannitol/PS of 1:2 leads to such a low content of mannitol at the surface (Figure 4d) that the granules do not retain their spherical shape after etching (Figure 4h).

The micrographs of the etched granules (Figure 4e–h) confirm that ethyl acetate dissolves the PS beads in the samples where they are accessible from the surface through an interconnected network (see above, discussion of the thermal analysis results).

The results in this section cannot be compared in detail with the work of Nandiyanto et al. [29] and Iskandar et al. [30] on hyaluronic acid (HA)/PS beads because these authors used smaller beads (100 nm, 200 nm, 300 nm), lower concentrations (hence smaller granules with a diameter typically below 1 µm) and different ways of varying the HA/PS ratios. When keeping the HA concentration constant and decreasing the PS bead content, Nandiyanto et al. observed a decrease in the number of surface pores [29]. This is coherent with our results, where we kept the PS concentration constant and increased the mannitol concentration. More generally, our results on the mannitol/PS system confirm their conclusion that the “spray-drying + etching of PS beads” strategy is not limited to inorganic matrix materials and can be successfully applied to the preparation of the porous particles of organic compounds.

### 3.5. Upscaled Production for the 1:1 Mannitol/PS Ratio

Based on the results discussed in the previous section, the 1:1 mannitol/PS ratio was considered as the most suitable to obtain spherical granules with a good homogeneity of porosity. Therefore, granules with a 1:1 mannitol/PS ratio were produced in larger quantity in a pilot-scale spray dryer equipped with a two-fluid nozzle. In order to maintain a granule size similar to the granules prepared with the lab-scale spray dryer, the experimental parameters had to be adapted (see Table 1 and Table 2, N-1/1-TF sample): a higher concentration of the feedstock solution was required to achieve efficient drying at the higher liquid flow rate and was compensated by the smaller droplet size resulting from the higher atomization flow rate. As expected, due to the larger drying chamber, the yield (50%) was improved with respect to the values obtained with the Büchi lab-scale spray dryer (25%). A higher yield could probably be obtained for larger feedstock volumes.

**Figure 7:**
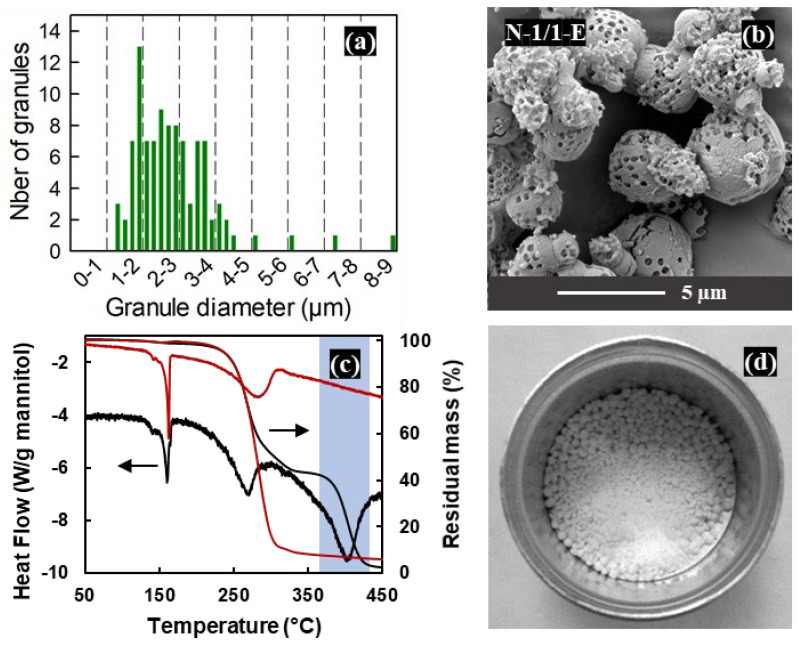
Upscaling of the synthesis for the M:PS ratio = 1:1 composition in the Niro pilot-scale spray dryer with the two-fluid nozzle: (**a**) histogram of the granule diameters, (**b**) SEM micrograph of the granules after etching, (**c**) TGA/DSC curves before (in black) and after (in red) etching, (**d**) photograph of the agglomerated formulation after mixing with the budesonide API.

The histogram of granule diameters in Figure 7a shows that the size range of the distribution is similar to the sample prepared in the lab-scale spray dryer (Figure 4k), although a larger proportion of granules seems to be found in the highest diameter zone of the range. This may explain why the distribution of the pores at the surface of the granules is less homogeneous (Figure 7b), while sufficient to ensure effective etching as seen in the thermal analysis curve (Figure 7c). Figure 5 shows that the shift to the pilot-scale spray dryer did not affect the fusion and decomposition temperatures of mannitol, or the percentage of β polymorph.

The pore distribution could have been optimized by fine-tuning the mannitol/PS ratio, possibly through a DoE (Design of Experiments) procedure. However, the preliminary tests of mixing the porous mannitol granules with the budesonide API revealed a severe agglomeration (Figure 7d), which would need to be solved before the porous mannitol granules could be considered for application as a fine porous excipient for DPI technology.

### 3.6. Larger Porous Granules Prepared with Rotary Nozzle Atomisation

As a complement to the experiments reported above for spray-drying conditions yielding granules with diameters < 5 µm, a solution with a 1:1 mannitol/PS ratio was also sprayed in the pilot-scale spray dryer equipped with a rotary nozzle. The larger droplet sizes and different trajectories in the drying chamber [51] offered by this atomization mode expand the panorama of possible microstructures for the 1:1 mannitol/PS ratio.

**Figure 8:**
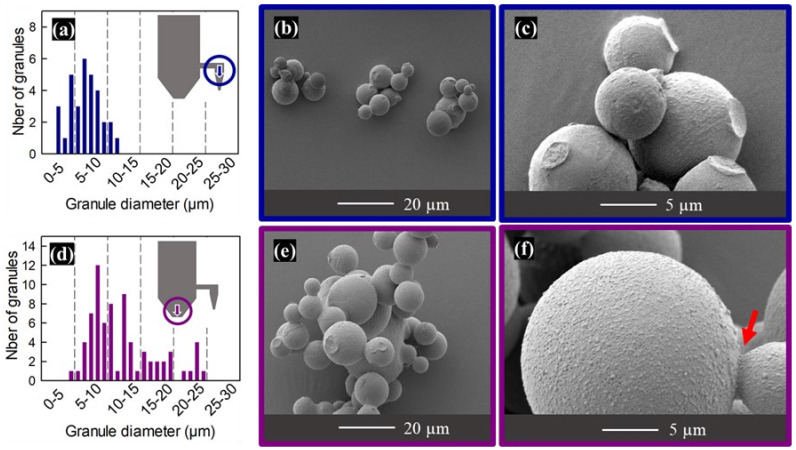
Histograms of granule diameters and SEM micrographs of as-sprayed granules prepared for the M:PS ratio = 1:1 composition in the Niro pilot-scale spray dryer with the rotary nozzle: (**a**–**c**) granules collected at the bottom of the cyclone (blue arrow on 8a); (**d**–**f**) granules collected at the bottom of the drying chamber (purple arrow on 8d). The red arrow highlights a neck between two granules.

The histograms and SEM micrographs in Figure 8 reveal a broad distribution of granule diameters, especially for the granules collected at the bottom of the drying chamber (between 4 and 25 µm—Figure 8d) when compared to the granules collected at the bottom of the cyclone (between 3 and 12 µm—Figure 8a). The overall yield was about 20%, from which about 70 wt% was recovered at the bottom of the cyclone.

**Figure 9:**
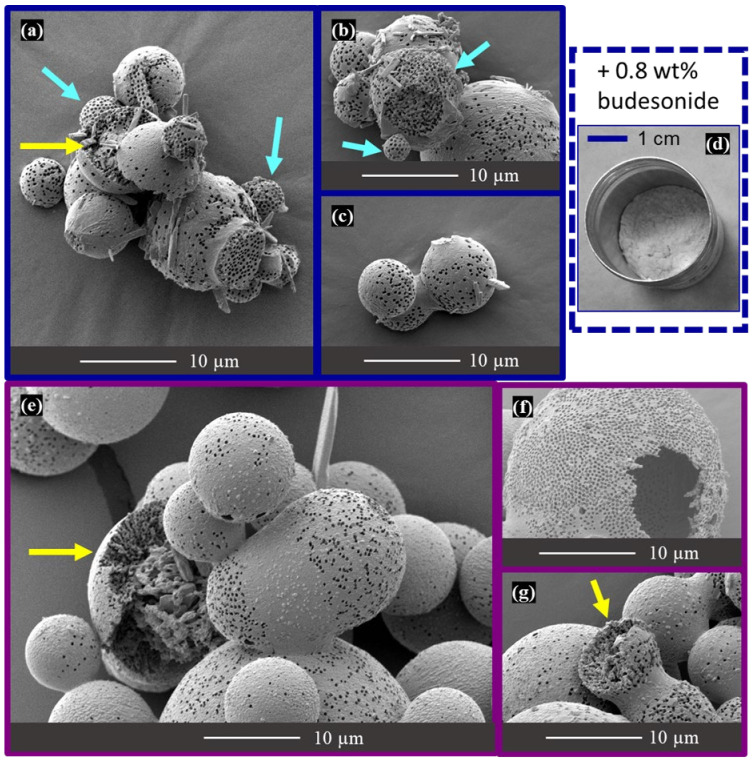
(**a**–**c**,**e**–**g**) The SEM micrographs of etched granules prepared for the M:PS ratio = 1:1 composition in the Niro pilot-scale spray dryer with the rotary nozzle; (**d**) photograph of the formulation after mixing with the budesonide API. Figures corresponding to granules collected at the bottom of the cyclone or at the bottom of the drying chamber are outlined in dark blue and dark pink, respectively. The arrows highlight examples of granule microstructures discussed in the text.

The micrographs of the etched granules in Figure 9 show that these spray-drying conditions result in a variety of microstructures and pore distributions. For the majority of the granules, only a fraction of their surfaces display open pores resulting from the etching of exposed PS spheres. As discussed in Section 3.4, the non-porous fraction of the surfaces probably corresponds to areas where the PS beads near the droplet surface were still impregnated by the solution when mannitol saturation was reached during spray drying [16,50]. A few broken spheres (marked with yellow arrows in Figure 9) reveal a thick, porous layer below the surface; the center of the largest amongst these spheres is occupied by a more disordered core including some large mannitol crystallites. A minority of granules, amongst the smallest ones, display a much more homogeneous distribution of surface pores (marked with light blue arrows in Figure 9) in agreement with the similar-sized granules obtained in the previous sections. Finally, a few large hollow granules, such as the one shown in Figure 9f, display open pores on their whole surface.

The SEM micrographs in Figure 8 and Figure 9 also reveal the presence of necks connecting the granules. The red arrow in Figure 8f points to one such neck, while several of the granules in Figure 8c,e display circular shallow craters that probably result from the breaking of such necks. Most of these observations suggest that these necks were created by the sticking together of still-wet granules and that the necks break easily to free the individual granules. However, in a few cases the degree of coalescence is more advanced, with the radius of the neck approaching that of the granules; an extreme case is that of the non-spherical granule in Figure 9e.

This microstructural inspection of the granules prepared with the rotary nozzle configuration confirms that larger droplet/granule sizes and the resultant change in surface/volume ratio require a modification of the mannitol/PS ratio to reach a good homogeneity of the distribution of the surface pores created by the etching of the exposed PS spheres. In its present state, the range of granule sizes prepared with the rotary nozzle configuration (3–25 µm) overlaps the ranges of fine (<10 µm) and coarse (20–100 µm) excipients. However, the fraction with the smaller granule diameters and narrower size distribution (i.e., the fraction collected at the bottom of the cyclone) was tested by mixing with 0.8 wt% budesonide API to check whether the severe agglomeration observed for the granules prepared in two-fluid mode would also be observed. As can be seen in Figure 9d, there was almost no agglomeration. Therefore, the budesonide content was measured by UHPLC in two aliquots taken from the formulation and values corresponding to 93.1% and 99.7% of the amount expected in the case of a perfectly homogeneous mixing were obtained. These preliminary tests are promising indications that the agglomeration problem could be solved and that the spray-dried porous granules could perform well as a porous fine excipient.

## 4. Conclusions

Porous mannitol granules with a diameter of about 2–3 microns were successfully produced by etching spray-dried composite granules of mannitol and polystyrene beads with ethyl acetate, an FDA approved solvent, in order to remove the polymer template. The lowest PS content (10:1 M:PS ratio) led to the encapsulation of most of the PS beads inside the mannitol matrix. Higher PS contents resulted in an open porous network after etching. In lab-scale conditions, the 1:1 M:PS ratio was found to provide the best homogeneity of porosity distribution, but the impact of other parameters (such as the droplet size) on the porosity distribution was evidenced when transferring to the pilot scale. The presence of the polystyrene beads in the spray-drying feedstock affected the polymorphism of mannitol (favoring the α polymorph) and its decomposition temperature in helium (decreasing by about 40 °C). The spray-drying configuration with a two-fluid nozzle yielded spherical porous mannitol granules with a diameter of about 2–3 microns (slightly larger for the pilot-scale system), which is in the suitable range for application as a porous fine excipient.

## Figures and Tables

**Table 1:** Overview of the M/PS granules obtained with different sets of synthesis conditions (M = mannitol, PS = polystyrene beads).

Label	M/PS Ratio (*w*/*w*)	Spray Dryer	(Mannitol) (g/L)	(PS Beads) (g/L)	Expected PS Content in Spray-Dried Granules	TGA-Measured PS Content in Spray-Dried Granules	T_melting_ for Mannitol
B-1/0	1/0	Büchi, Two-fluid nozzle	36	0	0 wt%	Not applicable	165
B-10/1	10/1	Büchi, Two-fluid nozzle	33.6	3.36	9 wt%	7.9 wt%	165
B-10/6	10/6	Büchi, Two-fluid nozzle	5.60	3.36	37.5 wt%	34.9 wt%	162
B-1/1	1/1	Büchi, Two-fluid nozzle	3.36	3.36	50 wt%	48.7 wt%	160
B-1/2	1/2	Büchi, Two-fluid nozzle	1.18	3.36	66.7 wt%	-	-
N-1/1-TF	1/1	Niro, Two-fluid nozzle	20.18	20.18	50 wt%	43.0 wt%	161
N-1/1-R	1/1	Niro, Rotary nozzle	20.18	20.18	50 wt%	-	162

**Table 2:** Experimental parameters for the syntheses in the lab- and pilot-scale spray dryers.

Spray-Drying Parameters	Büchi (Lab-Scale)	Niro (Pilot-Scale)	
	*Two-Fluid Nozzle*	*Two-Fluid Nozzle*	*Rotary Nozzle*
Inlet temperature (°C)	120	120	120
Outlet temperature (°C)	43	72	75
Atomization gas flow rate (m^3^/h)	0.6	6.7	6.7
Drying gas flow rate (m^3^/h)	29.4	67	67
Liquid flow rate (mL/min)	5	25	25
Injected volume (mL)	25	250	250
Typical experimental yield	25%	50%	20%

## Data Availability

The data presented in this study are available from the corresponding author, M.V., upon reasonable request.

## Supplementary Materials

The following supporting information can be downloaded at: https://www.mdpi.com/article/10.3390/ma16010025/s1, Figure S1: IR spectra before and after etching of PS beads (B-1/1 sample).

## References

[1] Zhou M., Shen L., Lin X., Hong Y., Feng Y. (2017). Design and Pharmaceutical Applications of Porous Particles. RSC Adv..

[2] Ahuja G., Pathak K. (2009). Porous Carriers for Controlled/Modulated Drug Delivery. Indian J. Pharm. Sci..

[3] Thananukul K., Kaewsaneha C., Opaprakasit P., Lebaz N., Errachid A., Elaissari A. (2021). Smart Gating Porous Particles as New Carriers for Drug Delivery. Adv. Drug Deliv. Rev..

[4] Saffari M., Ebrahimi A., Langrish T. (2016). A Novel Formulation for Solubility and Content Uniformity Enhancement of Poorly Water-Soluble Drugs Using Highly-Porous Mannitol. Eur. J. Pharm. Sci..

[5] Duddu S.P., Sisk S.A., Walter Y.H., Tarara T.E., Trimble K.R., Clark A.R., Eldon M.A., Elton R.C., Pickford M., Hirst P.H. (2002). Improved Lung Delivery from a Passive Dry Powder Inhaler Using an Engineered PulmoSphere?. Powder. Pharm. Res..

[6] Geller D.E., Weers J., Heuerding S. (2011). Development of an Inhaled Dry-Powder Formulation of Tobramycin Using Pulmosphere^TM^ Technology. J. Aerosol Med. Pulm. Drug Deliv..

[7] Dellamary L.A., Tarara T.E., Smith D.J., Woelk C.H., Adractas A., Costello M.L., Gill H., Weers J.G. (2000). Hollow Porous Particles in Metered Dose Inhalers. Pharm. Res..

[8] Xu S., Shi Y., Wen Z., Liu X., Zhu Y., Liu G., Gao H. (2023). Applied Catalysis B: Environmental Polystyrene Spheres-Templated Mesoporous Carbonous Frameworks Implanted with Cobalt Nanoparticles for Highly Efficient Electrochemical Nitrate Reduction to Ammonia. Appl. Catal. B Environ..

[9] Liu X., Verma G., Chen Z., Hu B., Huang Q., Yang H., Ma S., Wang X., Liu X., Verma G. (2022). Metal-Organic Framework Nanocrystal-Derived Hollow Porous Materials: Synthetic Strategies and Emerging Applications Metal-Organic Framework Nanocrystal-Derived Hollow Porous Materials: Synthetic Strategies and Emerging Applications. Innovation.

[10] Chen X., Zhu Z., Vargun E., Li Y., Saha P., Cheng Q. (2023). Atomic Fe on Hierarchically Ordered Porous Carbon towards High- Performance Lithium-Sulfur Batteries. J. Electroanal. Chem..

[11] Chen Y., Zhou M., Huang Y., Ma Y., Yan L., Zhou X., Ma X., Zhao X., Chen C., Bai J. (2022). Enhanced Ethanol Oxidation over Pd Nanoparticles Supported Porous Graphene-Doped MXene Using Polystyrene Particles as Sacrificial Templates. Rare Met..

[12] Shakeel A., Rizwan K., Farooq U., Iqbal S., Ali A. (2022). Chemosphere Advanced Polymeric/Inorganic Nanohybrids: An Integrated Platform for Gas Sensing Applications. Chemosphere.

[13] Gurung S., Gucci F., Cairns G., Chianella I., Leighton G.J.T. (2022). Hollow Silica Nano and Micro Spheres with Polystyrene Templating: A Mini-Review. Materials.

[14] Daem N., Mayer A., Spronck G., Colson P., Loicq J., Henrist C., Cloots R., Maho A., Dewalque J. (2022). Inverse Opal Photonic Nanostructures for Enhanced Light Harvesting in CH 3 NH 3 PbI 3 Perovskite Solar Cells. Appl. Nano Mater..

[15] Lv K., Zhang J., Zhao X., Kong N., Tao J., Zhou J. (2022). Understanding the Effect of Pore Size on Electrochemical Capacitive Performance of MXene Foams. Small.

[16] Alhajj N., O’Reilly N.J., Cathcart H. (2021). Designing Enhanced Spray Dried Particles for Inhalation: A Review of the Impact of Excipients and Processing Parameters on Particle Properties. Powder Technol..

[17] Vehring R. (2008). Pharmaceutical Particle Engineering via Spray Drying. Pharm. Res..

[18] Nandiyanto A.B.D., Okuyama K. (2011). Progress in Developing Spray-Drying Methods for the Production of Controlled Morphology Particles: From the Nanometer to Submicrometer Size Ranges. Adv. Powder Technol..

[19] Nandiyanto A.B.D., Ogi T., Wang W.N., Gradon L., Okuyama K. (2019). Template-Assisted Spray-Drying Method for the Fabrication of Porous Particles with Tunable Structures. Adv. Powder Technol..

[20] Gervelas C., Serandour A.L., Geiger S., Grillon G., Fritsch P., Taulelle C., Le Gall B., Benech H., Deverre J.R., Fattal E. (2007). Direct Lung Delivery of a Dry Powder Formulation of DTPA with Improved Aerosolization Properties: Effect on Lung and Systemic Decorporation of Plutonium. J. Control Release.

[21] Straub J.A., Chickering D.E., Lovely J.C., Zhang H., Shah B., Waud W.R., Bernstein H. (2005). Intravenous Hydrophobic Drug Delivery: A Porous Particle Formulation of Paclitaxel (AI-850). Pharm. Res..

[22] Kaplin I.Y., Lokteva E.S., Golubina E.V., Lunin V.V. (2020). Template Synthesis of Porous Ceria-Based Catalysts for Environmental Application. Molecules.

[23] Shchukin D.G., Caruso R.A. (2004). Template Synthesis and Photocatalytic Properties of Porous Metal Oxide Spheres Formed by Nanoparticle Infiltration. Chem. Mater..

[24] Zhang H., Hardy G.C., Khimyak Y.Z., Rosseinsky M.J., Cooper A.I. (2004). Synthesis of Hierarchically Porous Silica and Metal Oxide Beads Using Emulsion-Templated Polymer Scaffolds. Chem. Mater..

[25] Gradoń L., Janeczko S., Abdullah M., Iskandar F., Okuyama K. (2004). Self-Organization Kinetics of Mesoporous Nanostructured Particles. AIChE J..

[26] Iskandar F., Mikrajuddin, Okuyama K. (2001). In Situ Production of Spherical Silica Particles Containing Self-Organized Mesopores. Nano Lett..

[27] Balgis R., Ogi T., Arif A.F., Anilkumar G.M. (2014). Morphology Control of Hierarchical Porous Carbon Particles from Phenolic Resin and Polystyrene Latex Template via Aerosol Process. Carbon N. Y..

[28] Nandiyanto A.B.D., Hagura N., Iskandar F., Okuyama K. (2010). Design of a Highly Ordered and Uniform Porous Structure with Multisized Pores in Film and Particle Forms Using a Template-Driven Self-Assembly Technique. Acta Mater..

[29] Nandiyanto A.B.D., Okuyama K. (2017). Influences of Size and Amount of Colloidal Template and Droplet Diameter on the Formation of Porous-Structured Hyaluronic Acid Particles. Indones. J. Sci. Technol..

[30] Iskandar F., Nandiyanto A.B.D., Widiyastuti W., Young L.S., Okuyama K., Gradon L. (2009). Production of Morphology-Controllable Porous Hyaluronic Acid Particles Using a Spray-Drying Method. Acta Biomater..

[31] Rahimpour Y., Kouhsoltani M., Hamishehkar H. (2014). Alternative Carriers in Dry Powder Inhaler Formulations. Drug Discov. Today.

[32] Maas S.G., Schaldach G., Littringer E.M., Mescher A., Griesser U.J., Braun D.E., Walzel P.E., Urbanetz N.A. (2011). The Impact of Spray Drying Outlet Temperature on the Particle Morphology of Mannitol. Powder Technol..

[33] De Boeck K., Haarman E., Hull J., Lands L.C., Moeller A., Munck A., Riethmüller J. (2017). Inhaled Dry Powder Mannitol in Children with Cystic Fi Brosis: A Randomised Ef Fi Cacy and Safety Trial. J. Cyst. Fibros..

[34] Mönckedieck M., Kamplade J., Fakner P., Urbanetz N.A., Walzel P., Steckel H., Scherließ R. (2017). Dry Powder Inhaler Performance of Spray Dried Mannitol with Tailored Surface Morphologies as Carrier and Salbutamol Sulphate. Int. J. Pharm..

[35] Littringer E.M., Mescher A., Schroettner H., Achelis L., Walzel P., Urbanetz N.A. (2012). Spray Dried Mannitol Carrier Particles with Tailored Surface Properties—The Influence of Carrier Surface Roughness and Shape. Eur. J. Pharm. Biopharm..

[36] Jones M.D., Price R. (2006). The Influence of Fine Excipient Particles on the Performance of Carrier-Based Dry Powder Inhalation Formulations. Pharm. Res..

[37] Chow M.Y.T., Qiu Y., Lo F.F.K., Lin H.H.S., Chan H.K., Kwok P.C.L., Lam J.K.W. (2017). Inhaled Powder Formulation of Naked SiRNA Using Spray Drying Technology with L-Leucine as Dispersion Enhancer. Int. J. Pharm..

[38] Arzi R.S., Sosnik A. (2018). Electrohydrodynamic Atomization and Spray-Drying for the Production of Pure Drug Nanocrystals and Co-Crystals ☆. Adv. Drug Deliv. Rev..

[39] Walsh D., Serrano D.R., Marie A., Norris B.A. (2018). Production of Cocrystals in an Excipient Matrix by Spray Drying. Int. J. Pharm..

[40] Rowe R.C., Sheskey P.J., Quinn M.E., Rowe R.C., Sheskey P.J., Quinn M.E. (2009). Handbook of Pharmaceutical Excipients.

[41] Grodowska K., Parczewski A. (2010). Organic Solvents in the Pharmaceutical Industry. Acta Pol. Pharm. Drug Res..

[42] Dimian A.C., Bildea C.S., Kiss A.A. (2014). Chemical Product Design.

[43] Food and Drug Administration, Center for Drug Evaluation and Research Q3C—Tables and List Guidance for Industry Q3C—Tables and List Guidance for Industry. https://www.fda.gov/media/71737/download.

[44] Food and Drug Administration, Center for Drug Evaluation and Research Appendix 6. Toxicological Data For Class 3 Solvents. https://www.fda.gov/regulatory-information/search-fda-guidance-documents/q3c-appendix-6.

[45] Hartwig A.M.C. (2019). Ethyl Acetate Ethyl Acetate. MAK Collect. Occup. Heal. Saf..

[46] Yohanala P.T.F., Mulya Dewa R., Quarta K., Widiyastuti W., Winardi S. (2015). Preparation of Polystyrene Spheres Using Surfactant-Free Emulsion Polymerization. Mod. Appl. Sci..

[47] Cai Z., Teng J., Yan Q., Zhao X.S. (2012). Solvent Effect on the Self-Assembly of Colloidal Microspheres via a Horizontal Deposition Method. Colloids Surfaces A Physicochem. Eng. Asp..

[48] Cheary B.Y.R.W., Coelho A. (1992). A Fundamental Parameters Approach to X-Ray Line-Profile Fitting. J. Appl. Crystallogr..

[49] Fang J., Xuan Y., Li Q. (2010). Preparation of Polystyrene Spheres in Different Particle Sizes and Assembly of the PS Colloidal Crystals. Sci. China Technol. Sci..

[50] Handscomb C.S., Kraft M., Bayly A.E. (2009). A New Model for the Drying of Droplets Containing Suspended Solids after Shell Formation. Chem. Eng. Sci..

[51] Zafiryadis F.L. (2019). Numerical Modeling of Droplet Trajectories in Pilot Plant Spray Dryer Fitted with Rotary Atomizer. Masters’ Thesis.

