# Supplementary material for: Macroporous Mannitol Granules Produced by Spray Drying and Sacrificial Templating
Source: Materials (Basel). 2022 Dec 21;16(1):25. doi: 10.3390/ma16010025 (PMC9821148; doi:10.3390/ma16010025)
Supplement: Supplementary file 1 [file materials-16-00025-s001.zip › materials-2082763-supplementary.pdf]

Supplementary material

"Spray-drying of microporous granules of mannitol excipient using polystyrene beads as sacrificial template"

Morgane Valentin[1*], Damien Coibion[1], Bénédicte Vertruyen[1], Cédric Malherbe[2], Rudi Cloots[1], Frédéric Boschini[1]

[1] Group of Research in Energy and Environment from Materials, CESAM Research Unit, University of Liège, Liège 4000, Belgium

[2] Mass Spectrometry Laboratory, MolSys Research Unit, University of Liège, Liège 4000, Belgium

*Corresponding authors. Email address : morgane.valentin@uliege.be (M.V.), b.vertruyen@uliege.be (B.V.)

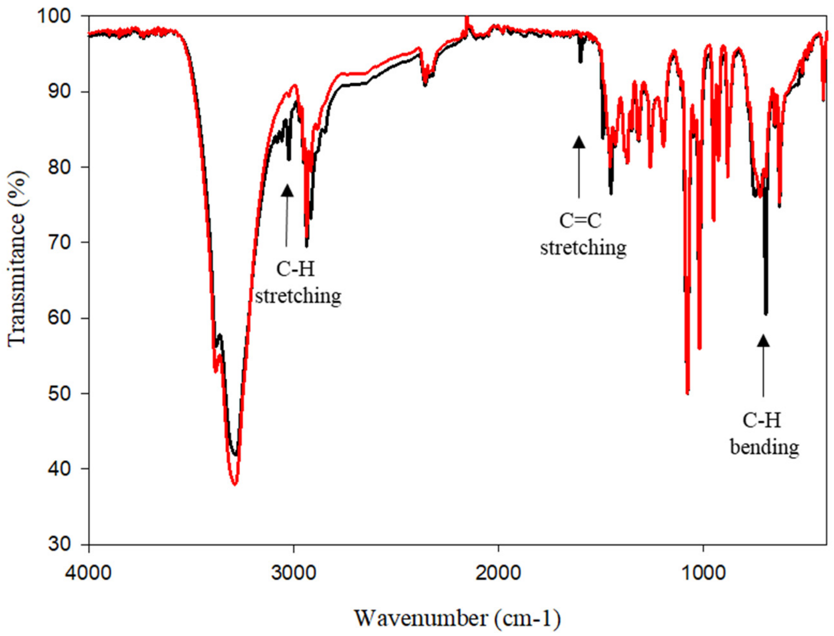

Figure S1. IR spectra before (black curve) and after (red curve) etching of PS beads (B-1/1 sample)
